# Mortality following atypical femoral fractures in men

**DOI:** 10.1093/jbmr/zjag036

**Published:** 2026-02-23

**Authors:** Colton Hoffer, William Obremskey, Binni Makkar, Brinda Basida, Nitya Shah, Jared D Huling, Zhiping Huo, Emily Budde, Reside Jacob, John T Schousboe, Howard A Fink, Robert A Adler, Joan C Lo, Ryan Avidano, Joshua I Barzilay, Frances Weaver, Susan M Ott, KinhTu Lien, Laura D Carbone

**Affiliations:** Department of Medicine, Division of Rheumatology, Medical College of Georgia at Augusta University, Augusta, GA 30912, United States; Department of Orthopedics, Vanderbilt University, Nashville, TN 37240, United States; Department of Medicine, Division of Rheumatology, Medical College of Georgia at Augusta University, Augusta, GA 30912, United States; Department of Medicine, Division of Rheumatology, Medical College of Georgia at Augusta University, Augusta, GA 30912, United States; Division of Biostatistics and Health Data Science, University of Minnesota School of Public Health, Minneapolis, MN 55455, United States; Division of Biostatistics and Health Data Science, University of Minnesota School of Public Health, Minneapolis, MN 55455, United States; Edward Hines Jr. Veterans Affairs Medical Center, Veterans Affairs Health Care System, Chicago, Illinois 60141, United States; Edward Hines Jr. Veterans Affairs Medical Center, Veterans Affairs Health Care System, Chicago, Illinois 60141, United States; Edward Hines Jr. Veterans Affairs Medical Center, Veterans Affairs Health Care System, Chicago, Illinois 60141, United States; Division of Research, Park Nicollet Clinic and HealthPartners Inc., Saint Paul, MN 55114, United States; Geriatric Research Education and Clinical Center, VA Health Care System, Minneapolis, MN 55417, United States; Endocrinology and Metabolism Section, Richmond Veterans Affairs Medical Center, Richmond, VA 23249, United States; Division of Research, Kaiser Permanente Northern California, Pleasanton, CA 94588, United States; Department of Medicine, Division of Rheumatology, Medical College of Georgia at Augusta University, Augusta, GA 30912, United States; Division of Endocrinology, Kaiser Permanente of Georgia, Emory University School of Medicine, Atlanta, GA 30308, United States; Edward Hines Jr. Veterans Affairs Medical Center, Veterans Affairs Health Care System, Chicago, Illinois 60141, United States; Department of Medicine, University of Washington, Seattle, WA 98195, United States; Department of Medicine, Division of Rheumatology, Medical College of Georgia at Augusta University, Augusta, GA 30912, United States; Department of Medicine, Division of Rheumatology, Medical College of Georgia at Augusta University, Augusta, GA 30912, United States; Edward Hines Jr. Veterans Affairs Medical Center, Veterans Affairs Health Care System, Chicago, Illinois 60141, United States

**Keywords:** osteoporosis, atypical femoral fractures, hip fractures, mortality, bisphosphonates

## Abstract

Atypical femoral fractures (AFFs) are an uncommon complication of bisphosphonate (BP) treatment for osteoporosis. The purpose of this report was to determine differences in mortality among men treated with BPs who experienced AFFs, hip fractures, or typical subtrochanteric/femoral shaft (ST/FS) fractures. We included men from the Veterans Affairs Corporate Data Warehouse who filled at least one BP prescription over a nearly 20-yr span (October 1, 1999, to December 31, 2022). Typical ST/FS and hip fractures after a first filled prescription for a BP were identified using ICD codes. Radiographic review was conducted for ST/FS fractures to determine which were AFFs using the American Society of Bone and Mineral Research 2014 criterion. Demographic and clinical characteristics and cumulative duration of BP exposure prior to the incident fracture were determined. There were 23 men with an AFF, 4591 with a hip fracture, and 225 with a (non-AFF) ST/FS fracture. At 12-mo post-fracture, 4% of men with an AFF had died, compared with 25% of men with a hip fracture and 15% of men with an ST/FS fracture. Over 5 yrs. of follow-up, mortality following fracture differed among the 3 different fracture groups (χ^2^ = 6.21, d.f. = 2, *p* = .045) with death occurring in 7 of 23 (30%) men with an AFF, 2380 of 4591 (52%) men with a hip fracture and 109 of 225 (48%) men with a ST/FS fracture. In Cox proportional hazard models, over 5 yrs, with and without adjustment for covariates, there were no significant differences in mortality following an AFF compared with a hip fracture or an AFF compared with a ST/FS fracture (*p* > .10 for all). It is possible that mortality is lower following AFF compared with a hip or ST/FS fracture; however, limited sample size among those with AFF may have precluded ascertainment of such a difference.

## Introduction

Osteoporotic fractures cause substantial morbidity, increased mortality, and loss of independence, and incur significant societal economic burdens among older individuals.^1^ Hip fractures in particular are associated with excess mortality, with 1-yr mortality following a hip fracture among adults in the United States (U.S.) older than age 60 yr approximating 21%.^2^ Mortality following subtrochanteric and femoral shaft fractures is also substantial, with 1 yr mortality rates ranging from 17%^3^ to 25%.^4^

Randomized clinical trials demonstrate that oral or intravenous (IV) bisphosphonate (BP) therapy following a hip or vertebral fracture substantially reduces subsequent fractures.^5,6^ Despite this, there has been a continuous decline in osteoporosis drug initiation rates, with one study reporting a precipitous decline in these rates from 9.8% in 2004 to 3.3% in 2015 among persons who had a hip fracture.^7^ Veterans Affairs prescribing patterns for BPs over this time mirrored national trends.^8^ In the face of these declining treatment rates, after consistent declines in fracture rates from 2007 to 2013, trends from 2014 to 2017 suggested that fracture rates may no longer be declining and, for some fractures, may be rising.^9^ Uncommon and late adverse events, such as atypical femoral fractures (AFFs),^10^ have contributed to a substantial concern related to suboptimal use of fracture-prevention therapies in populations at high risk for fracture.^11^

Atypical femoral fractures are an uncommon but serious late adverse effect from therapy with BPs, principally when used for skeletal health reasons and not cancer.^12^ Although most,^13–18^ but not all^19^ studies report that there is no increased mortality following AFFs compared with typical subtrochanteric and femoral shaft (ST/FS) fractures,^20^ a recent systematic review suggested that there is insufficient data to determine the relationship of AFFs to mortality.^21^

Moreover, the majority of reports examining the association of AFFs with mortality among users of BPs have little to no data on men (2 studies with 4 men,^16,22^ 1 study with 6 men,^18^ 1 study with 7 men,^14^ and 1 study with 9 men^13^). The largest study to date reporting on the relationship of AFFs to mortality in men included 12 men with AFFs; however, only 4 of these men were BP users.^20^ Understanding the relationship of AFFs to mortality in men is important as following either a typical hip fracture,^23^ or a femoral shaft fracture,^3^ mortality is higher in men than women. Including non-White individuals is also important, as following hip^24^ and femur fractures,^25^ it has been reported that Black individuals have increased 1 yr mortality compared to those who are White.^24^

The purpose of this report was to examine mortality following AFFs compared with typical osteoporotic hip fractures and typical ST/FS fractures in a cohort of male Veterans who had a filled BP prescription for fracture prevention (and not for cancer-related reasons) prior to the fracture.

## Materials and methods

### Inclusion criteria

The analysis cohort included all male Veterans aged 50 yr and older with data in the VA Corporate Data Warehouse (CDW) who had at least one filled prescription for an FDA-approved oral or IV BP for osteoporosis from October 1,1999 through December 31, 2022 prior to a principal inpatient International Classification of Diseases (ICD) code for a ST/FS fracture (ICD-9 codes 820.22, 820.32, 821.0, 821.1, 733.15, and 733.97, and ICD-10 codes S72.2, S72.3, M84.451, M84.452, M84.453, M84.351, M84.352, M84.353, and M84.75) or hip fracture ICD 9 (820.xx); ICD 10 (S72.0, S72.1, S72.2, M84.459, and M84.659) at the VHA. Men with a history of secondary metastatic cancer (ICD-9 codes 197X, 198.X, and 199.0; ICD-10 codes C78.X, C79.X, and C80.X), multiple myeloma (ICD-9 code 203.0X; ICD-10 code C90.0X), Paget’s disease (ICD-9 code 731.0; ICD-10 code M88.X), osteogenesis imperfecta (ICD-9 code 756.51; ICD-10 code Q78.0), hypophosphatasia or phosphate wasting syndromes (ICD-9 code 275.3; ICD-10 code E83.3X); osteopetrosis (ICD-9 code 756.52; ICD-10 code Q78.2), and end stage renal disease (ESRD) or renal failure (ICD-9 code 585.6; ICD-10 N18.6) were excluded. Those with a filled prescription at any time during the study period for etidronate, pamidronate, or tiludronate, and those whose second dose of zoledronic acid was ≤273 d from the initial dose (more frequent dosing intervals are used for cancer) were also excluded. Men who had received a prescription for teriparatide, denosumab, abaloparatide, or romosozumab at the time of or prior to the first hip (*n* = 32) or ICD coded ST/FS (*n* = 3) fracture were also excluded.

### Fracture ascertainment

All ST/FS ICD-coded fractures underwent independent radiographic review for features of AFF using 2014 American Society of Bone and Mineral Research (ASBMR) criteria (ie, that the fracture line originated at the lateral cortex and was substantially transverse in orientation, the fracture was noncomminuted or minimally comminuted, there was localized periosteal or endosteal thickening of the lateral cortex, and that complete fractures extended through both cortices while incomplete fractures involved only the lateral cortex^10^) by an orthopedic surgeon (WO). If the radiograph was deemed not to be an AFF, it was considered a typical ST/FS fracture. We also examined whether there were major trauma codes ICD-9 (E800-E848) and ICD-10 (V01-V99) within a 3-d period of the AFF.

The study was declared exempt by the VA central IRB and was performed in accordance with the ethical standards as laid down in the 1964 Declaration of Helsinki and its later amendments.

### Patient characteristics

Patient characteristics were ascertained at the date or closest to the date of the incident fracture and included age, race, ethnicity, BMI, smoking status (current, past, never, and unknown), area deprivation index (ADI) (as a marker of socioeconomic status),^26^ and clinical fractures in the previous 5 yr (ICD-9 codes 733.1, 805.X., and 807-829.X; ICD-10 codes M48.4, M48.5, M80, M84.4, M84.6, S12.X, S22.X, S32.X, S43.X, S52.X, S62.X,S72.X, S82.X, and S92.X). Charlson comorbidity index (CCI)^27–30^ and Veterans Affairs frailty index (VA-FI) (nonfrail, prefrail, mildly frail, moderately frail, severely frail, or missing)^31^ (Table 1).

**Table 1:** Characteristics of men with AFFs, hip fractures, and subtrochanteric and typical subtrochanteric/femoral shaft fractures.

**Characteristic**	**AFF (*N* = 23)**	**Hip fracture (*N* = 4591)**	**Typical subtrochanteric and femoral shaft fractures (ST/FS) (*N* = 225)**	***p*-value**
**Age in years at fracture diagnosis date (mean ± SD) (median)**	(75 ± 9) (73)	(78 ± 10) (79)	(74 ± 11) (74)	<.001^*^
**Race**				
**White *N* (%)**	19 (83)	3886 (85)	187 (83)	.2
**Black *N* (%)**	3 (13)	287 (6.3)	24 (11)	.041^*^
**Asian *N* (%)**	0 (0)	14 (0.3)	0 (0)	.6
**Native Hawaiian/Pacific Islanders *N* (%)**	0 (0)	32 (0.7)	0 (0)	.5
**American Indian/Alaskan Native *N* (%)**	0 (0)	10 (0.2)	1 (0.4)	.4
**Multiracial *N* (%)**	0 (0)	33 (0.7)	2 (0.9)	.6
**Ethnicity**				.9
**Hispanic *N* (%)**	1 (4.3)	270 (5.9)	13 (5.8)	
**Non-Hispanic *N* (%)**	21 (91)	3914 (85)	192 (85)	
**Unknown *N* (%)**	1 (4.3)	407 (8.9)	20 (8.9)	
**Body mass index (kg/m** ^**2**^ **)**				.021^*^
**Underweight (<18.5) *N* (%)**	0 (0)	372 (8.2)	17 (7.6)	
**Healthy (18.5-24.9) *N* (%)**	8 (35)	2106 (46)	85 (38)	
**Overweight (25-29.9) *N* (%)**	9 (39)	1452 (32)	76 (34)	
**Obese (≥30) *N* (%)**	6 (26)	631 (14)	45 (20)	
**Unknown *N* (%)**	0 (0)	30 (0.7)	2 (0.9)	
**Smoking status**				.6
**Current *N* (%)**	6 (27)	1237 (30)	67 (32)	
**Past *N* (%)**	6 (27)	1433 (34)	78 (37)	
**Never *N* (%)**	10 (45)	1511 (36)	66 (31)	
**Unknown *N* (%)**	1 (4.3)	410 (8.9)	14 (6.2)	
**Area deprivation index (*n* = 2559) (mean ± SD) (Median Q1, Q3) (Min-Max)**	(*n* = 16) (51 ± 28) (57 32,68) (8-100)	(*n* = 2425) (55 ± 26) (57 37,76) (1-100)	(*n* = 118) (55 ± 27) (57 34,76) (1-100)	.7
**Charlson comorbidity index (mean ± SD) (median)**	(2.43 ± 2.35) (2.00)	(2.18 ± 2.03) (2.00)	(2.31 ± 2.07) (2.00)	.5
**VA Frailty Index**				.7
**NonFrail *N* (%)**	1 (4.3)	352 (7.7)	22 (9.8)	
**Prefrail *N* (%)**	4 (17)	988 (22)	42 (19)	
**Mildly frail *N* (%)**	8 (35)	1263 (28)	59 (26)	
**Moderately frail *N* (%)**	3 (13)	990 (22)	53 (24)	
**Severely frail *N* (%)**	7 (30)	998 (22)	49 (22)	
**Prevalent all clinical** ***fractures N* (%)**	11 (48)	1928 (42)	114 (51)	.032^*^

^*^Statistically significantly different among the groups.

### Bisphosphonate exposure

Cumulative days’ supply of BP from the initial and subsequent prescriptions were used to define exposure. We allowed stockpiling for up to a 30-d period. In the case of prescription overlap in excess of 30 d, we considered the first BP to have been stopped when the second BP was prescribed, and the second BP took precedence.^32^ We considered a single infusion of zoledronic acid to last for 18 mo for BP exposure definitions (Table 2).

**Table 2:** Bisphosphonate exposure prior to index fracture.

**Drug information**	**AFF (*N* = 23)**	**Hip fractures (*N* = 4591)**	**Typical subtrochanteric and femoral shaft fractures (ST/FS) (*N* = 225)**	***p*-value**
**Cumulative time of all BP exposure (months) (mean ± SD) (median)**	(69 ± 47) (64)	(51 ± 51) (35)	(45 ± 48) (28)	.019^*^
**Filled BP prescriptions *N* (%)^a^**				
**Alendronate**	22 (96)	4276 (93)	209 (93)	
**Risedronate**	0 (0)	238 (5.2)	16 (7.1)	
**Ibandronate**	0 (0)	8 (0.2)	1 (0.4)	
**Zoledronic acid**	3 (13)	345 (7.5)	18 (8)	

^a^Drug groups are not independent of each other. Patients may have been prescribed more than one BP over time. ^*^Statistically significantly different among the groups.

### Mortality ascertainment

Date of death was determined using the VA death ascertainment files^33^ (accessed August 27, 2024). This file includes data from a number of external and internal VA sources to comprehensively report death. External sources include the Social Security Administration (SSA), Death Master File (DMF), the National Death Index (NDI), the Centers for Medicare and Medicaid Services Medicare Vital Status File, and state death certificates. Internal VA data sources include the Beneficiary Identification and Records Location Subsystem (BIRLS), the Medical SAS Inpatient Datasets (MSID), the VA Master Index (VAMI), Patient Treatment Files (PTF), and the VA Mortality Data Repository (MDR).

Follow-up for death was a maximum of 5 yr, with mortality assessed at 0, 1, 2, 3, and 6 mo and yearly up to 5 yr following the incident fracture.

### Post-fracture care

To determine if there were differences in post-fracture care among the men with an AFF, hip, or ST/FS fracture, we analyzed median length of hospital stay, timing of hospital discharge (day of week of discharge) and duration of post-fracture rehabilitation at the VHA. Rehabilitation services were ascertained using VA Stop codes 205 (Physical Therapy), 206 (Kinesiotherapy), and 209 (Geriatric Physical Therapy).

### Statistical analysis

Characteristics of men with AFFs, hip fractures, and typical ST/FS fractures were compared using chi-squared tests for categorical variables and *t*-tests for continuous variables. Mortality by fracture group was examined using a Kaplan–Meier curve and compared across the 3 groups using a log-rank test. Due to violations of the proportional hazards assumption, a Cox proportional hazards (CPH) model allowing for time-dependent effects was used to examine the relationship between fracture group and mortality risk. Violations of proportional hazards occurred in most key adjustment covariates in addition to across AFF, hip fractures, and ST/FS groups, indicating that the hazard ratio from a standard CPH model could yield misleading conclusions. Violations of proportional hazards were detected using the testing procedure of Grambach and Therneuau.^34^ We used a CPH model with time-varying effects which allows the hazard ratios to vary over time and was adjusted for age at the time of fracture, cumulative BP exposure, presence of other prevalent fractures, race, ethnicity, smoking status, BMI, ADI, CCI, and VA-FI. Missing data for race, ethnicity, BMI, and smoking status were imputed using multiple imputation by chained equations (MICE) with predictive mean matching.^35^ Time-varying hazard ratios are reported through 5 yr of follow-up. The final estimates were pooled according to Rubin’s rules to account for uncertainty due to imputation and data missingness.^36^ All time-to-event analyses censored all observations at 5 yr of follow-up time. In post hoc analyses, we tested for differences in median length of hospitalization with Kruskal–Wallis tests, timing of discharge following the incident fracture (by day of the week of discharge) using Mantel–Haenszel Chi-square tests, and duration of rehabilitation at 3 and 12 mo following the incident fracture by Kruskal–Wallis tests. All analyses were conducted using R statistical computing language version 4.4.1.

## Results

**Figure 1:**
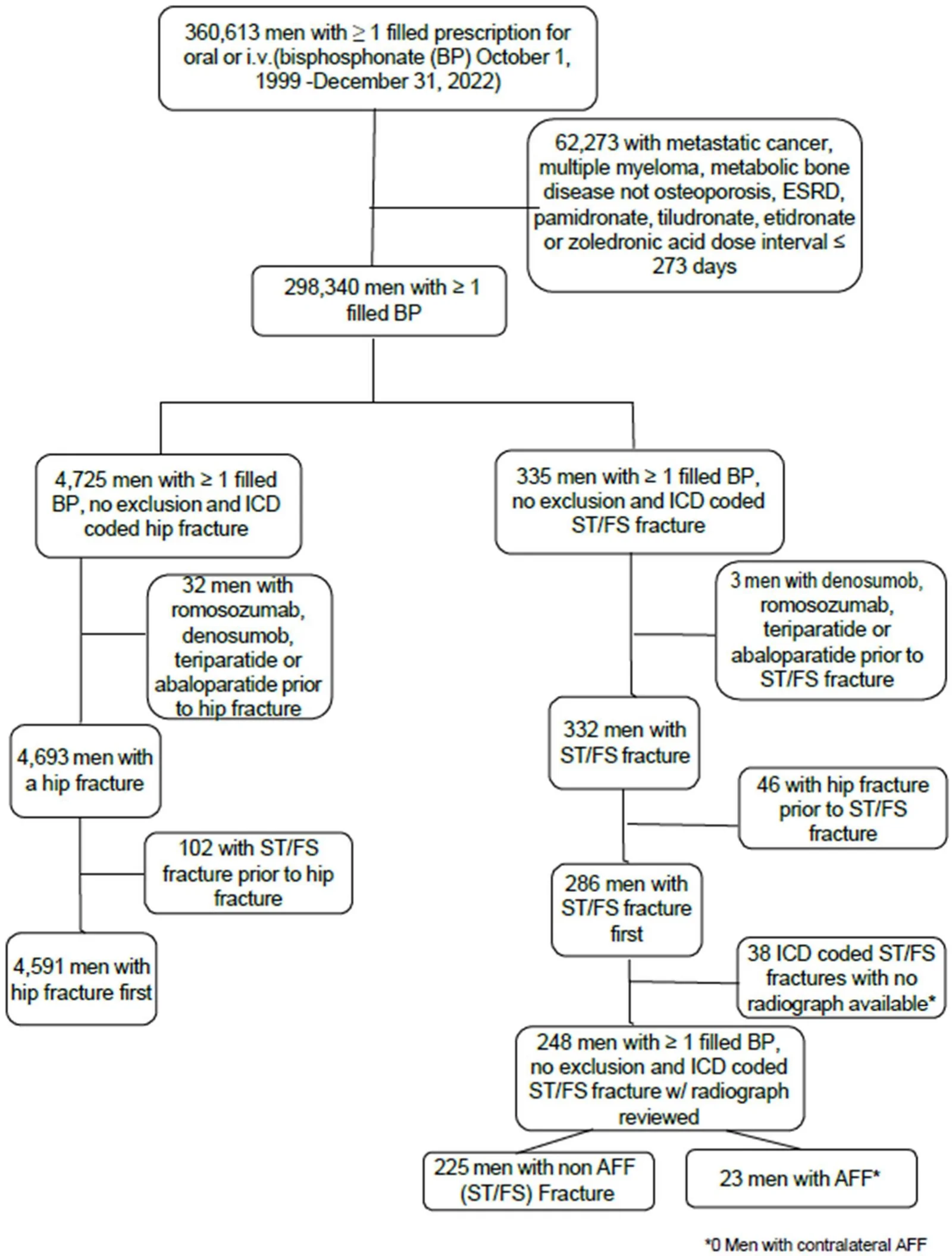
Flow diagram of study population.

There were 298 340 men with a least one filled prescription for an oral or IV BP who did not have a study exclusion over the course of the study. Figure 1 depicts the flow diagram of the study population of men with first incident hip fractures and men with first incident ST/FS fractures. If both a hip and a ST/FS fracture occurred, only the first of these fractures was considered. There were 102 men with a ST/FS fracture prior to a hip fracture who were included in the ST/FS fracture group and 46 men with a hip fracture prior to the ST/FS fracture who were only included in the hip fracture group.

Overall, 286 men had an ICD code for a first ST/FS fracture. Radiographs were not available for 38 ST/FS-coded fractures (13%), and they were excluded from analyses. Of those with available radiographs, 225 men had an ST/FS fracture identified by radiographic review and 23 men with an AFF. Seventy-eight percent of the AFFs were complete fractures. Three AFFs occurred in Black men. There were no men with a major trauma code ICD 9 (E800-E848) or ICD 10 (V01-V99) within a 3-d period of the AFF.

The demographic and clinical characteristics of the study population of 4839 men at the time of the fracture are shown in Table 1, comprising 4591 with first hip fracture, 225 with first ST/FS fracture (confirmed non-AFF), and 23 with AFF. The mean cumulative time of exposure to BPs was 69 mo (5.8 yr) for men with AFFs, 51 mo (4.3 yr) for men with hip fractures, and 45 mo (3.8 yr) for men with ST/FS fractures (*p* = .019). Alendronate was the most frequently prescribed oral BP **(**Table 2**)**.

**Figure 2:**
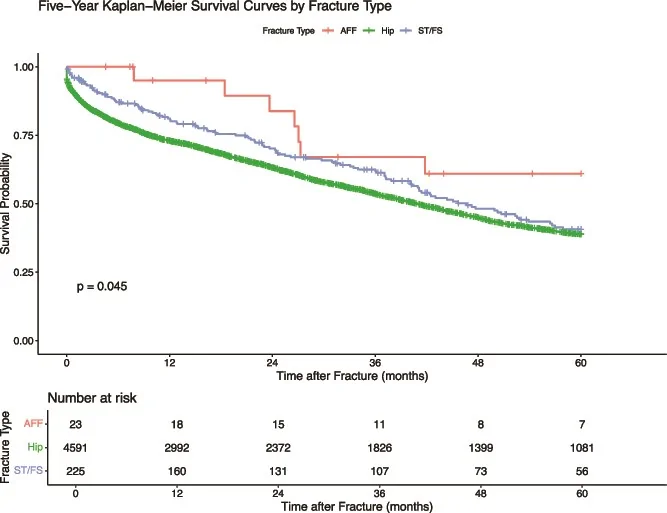
Kaplan–Meier survival curves by type of fracture.

Mortality at 1- and 5-yr post-fracture by fracture type is shown in Table 3. Kaplan Meier curves showing survival probability by fracture type are shown in Figure 2. At 12-mo post-fracture, 4% of men with an AFF had died, compared with 25% of men with a hip fracture and 15% of men with an ST/FS fracture. Over 5 yr of follow-up, mortality following fracture differed among the 3 different fracture groups (χ^2^ = 6.21, d.f. = 2, *p* = .045) with death occurring in 7 of 23 (30%) men with an AFF, compared to 2380 of 4591 (52%) men with a hip fracture and 109 of 225 (48%) men with a ST/FS fracture. However, in CPH models, there were no significant differences in terms of the unadjusted hazard ratios for mortality in men with AFFs compared with hip fractures or men with AFFs compared with ST/FS fractures (Table 4). Thus, differences in mortality between ST/FS and hip fractures likely drive the significant log-rank test result.

**Table 3:** Mortality at 1 and 5 yr for men by fracture type.

**Fractures** **(*N* = 4839)**	**AFF** **(*N* = 23)**	**Hip fractures** **(*N* = 4591)**	**Subtrochanteric or femoral shaft fracture (ST/FS)** **(*N* = 225)**
**Died at 1 yr** ***N* (%)**	1 (4.35)	1188 (25.88)	40 (17.77)
**Died at 5 yr** ***N* (%)**	7 (30.43)	2380 (51.84)	109 (48.44)

**Table 4:** Unadjusted mortality hazard ratios following fracture in men.

**Time (mo)**	**HR** **(AFF vs hip)**	**Lower 95% CI** **(AFF vs hip)**	**Upper 95% CI (AFF vs hip)**	**HR** **(AFF vs ST/FS)**	**Lower 95% CI** **(AFF vs ST/FS)**	**Upper 95% CI (AFF vs ST/FS)**
**0**	0.015	0.000101	2.223	0.057	0.000364	8.852
**1**	0.109	0.009	1.27	0.202	0.017	2.397
**2**	0.162	0.023	1.158	0.259	0.036	1.89
**3**	0.204	0.038	1.106	0.3	0.054	1.657
**6**	0.304	0.088	1.053	0.387	0.11	1.363
**12**	0.453	0.188	1.093	0.5	0.204	1.227
**24**	0.677	0.322	1.425	0.645	0.299	1.393
**36**	0.856	0.375	1.956	0.749	0.319	1.761
**48**	1.011	0.394	2.595	0.833	0.315	2.202
**60**	1.151	0.4	3.306	0.905	0.306	2.679

There were no significant differences in terms of hazard ratios for mortality adjusted for all covariates in men with AFFs compared with hip fractures (Table 5) or men with AFFs compared with ST/FS fractures (Table 6), possibly due to the small sample size and number of mortality events in the AFF group.

**Table 5:** Adjusted mortality hazard ratios following fracture in men with AFF vs hip fractures.

**Time (mo)**	**Hazard ratio**	**Lower 95% CI**	**Upper 95% CI**
**0**	0.0142	0.0000693	2.91
**1**	0.118	0.00875	1.58
**2**	0.178	0.0223	1.43
**3**	0.228	0.0385	1.35
**6**	0.349	0.0955	1.27
**12**	0.534	0.218	1.31
**24**	0.817	0.387	1.72
**36**	1.05	0.453	2.43
**48**	1.25	0.475	3.3
**60**	1.44	0.481	4.28

**Table 6:** Adjusted mortality hazard ratios following fracture in men with AFF vs ST/FS fractures.

**Time (mo)**	**Hazard ratio**	**Lower 95% CI**	**Upper 95% CI**
**0**	0.0439	0.000205	9.42
**1**	0.181	0.0132	2.49
**2**	0.24	0.0295	1.95
**3**	0.283	0.0469	1.71
**6**	0.376	0.101	1.39
**12**	0.5	0.2	1.25
**24**	0.665	0.307	1.44
**36**	0.787	0.331	1.87
**48**	0.886	0.327	2.4
**60**	0.971	0.316	2.98

The median length of hospitalization was 6.5 d for men with an AFF, 8 d for men with a hip fracture, and 8 d for men with a ST/FS fracture (*p* = .23). There were no significant differences in day of hospital discharge among the groups (*p* = .94). There were no significant differences in the number of rehabilitation visits at 3 (*p* = .78) or 12 (*p* = .72) mo post-fracture among the groups.

## Discussion

In this cohort of men treated with a BP for fracture prevention, survival for up to 5 yr following the index fracture was not significantly different in men with an AFF compared to men with a hip fracture or a typical ST/FS fracture. However, it is possible that mortality is lower following an AFF than a hip or ST/FS fracture, but small sample sizes of AFF may have precluded ascertainment of such a difference.

A number of studies have examined mortality rates at 1 yr post femur/hip fractures.^13,19,20,37^ In our cohort of men, we noted a 4% 1-yr post AFF mortality. In a Swedish study, there were no deaths following an AFF at 1 yr.^20^ A systematic review estimated a 10% mortality rate at 1 yr post AFF^21^; in contrast with these reports, our cohort was confined to men. Our finding of 15% mortality following an ST/FS at 1 yr is in line with previous reports of post-fracture mortality following these fractures, with 1 yr mortality rates ranging from 17%^3^ to 25%,^4^ again, in contrast with these studies, our cohort was confined to men. In accord with previous reports of substantial mortality following a hip fracture,^20,38–40^ particularly for men,^38–40^ 25% of men with a hip fracture in our cohort died in the first year following the fracture.

In the short-term period following the fracture, no men with an AFF died in the first 3 mo post-fracture compared to 10% of men with a hip fracture dying at 30 d post-facture and 15% at 90 d post-fracture. These post-hip fracture mortality rates are consistent with prior reports which estimate mortality at 9%-10% (30-d mortality), and 20%-23% (90-d mortality),^39,41^ but are, to our knowledge, the first report of short-term mortality following an AFF in men.

Others have reported no differences in overall mortality in patients with an AFF compared to those with a typical femoral fracture.^13–18^ In contrast, a Swedish study reported that mortality was much lower for men and women with AFFs (23%) compared to non-AFF fractures (62%) at a mean follow-up of 4 yr.^20^ Further, a study conducted in Singapore, including men and women with an AFF, reported higher 5-yr survival in those with AFFs compared with typical subtrochanteric fracture patients.^13^ In a report from Austria, median survival following an AFF was 9 mo compared to 18 mo for a typical femoral fracture.^19^ In view of these conflicting data, a recent systematic review concluded that there was insufficient evidence to conclude whether there was a difference in mortality risk between AFF and typical femoral fractures.^21^ Notably, our dataset which included 23 men with AFF, is, to our knowledge, the largest cohort confined to men with AFFs in the U.S. treated with BPs for fracture prevention, examined for comparisons with mortality to ST/FS fractures.

Compared to men with a hip or ST/SF fracture, men with an AFF were younger. The younger men with AFFs may have been healthier than the men with hip or ST/FS fractures in ways that were not captured. For example, we did not have a measure for time spent walking or nature of exercise. The AFF fractures have some features in common with stress fractures, in that micro-damage accumulates faster than it can be repaired. One study of young men in military training showed an increased rate of stress fractures in those randomly treated with a BP compared to placebo, although this was nonsignificant due to high drop-out rate.^42^ In addition to being younger, fewer men with an AFF were underweight or current smokers and were more often Black. In analyses adjusted to account for these and other differences in patient characteristics, hazard ratios consistently revealed a lower mortality risk for the AFF group. However, due to the limited statistical power inherent to the sample size of the AFF group, these differences were not significant. We also investigated similar Cox analyses without covariate adjustment, and the hazard ratios were similar to the adjusted hazard ratios, with similar confidence intervals, suggesting that sample size, rather than covariate adjustment, may be driving these findings.

Our findings that length of hospitalization, timing of hospital discharge, and number of post-fracture rehabilitation visits did not differ among the groups is in line with our findings relative to lack of mortality differences among these men with AFF, hip, and ST/FS fractures. However, although not statistically significantly different, the median length of hospitalization was much shorter in men with AFF compared to the other fracture groups, suggesting that we might have seen significant differences here had sample sizes of AFF been larger.

Our study has a number of important strengths. The inclusion of 23 men who had an AFF following a BP prescription for fracture prevention, although small, is, to our knowledge, the largest study of post-AFF mortality in men who had BP usage. Prior to our report, the largest study of AFF mortality in male BP users only included 9 men and was confined to patients in a single center in Singapore.^13^ In addition, our ascertainment of mortality at the VHA is comprehensive, as the new Death Ascertainment Files Data Source Library utilizes a number of validated sources to capture mortality.^43^ Further, our report is the first to examine mortality including multiple races. This is potentially important, because, at least for hip fractures, prior reports indicate that mortality in greater in Black individuals than White individuals.^44,45^ Atypical femoral fractures cannot be ascertained using administrative claims data; therefore, all fracture radiographs underwent independent blinded review by an orthopedic surgeon (W.O.).^46^ Ours is also the first study of post AFF mortality to include a social construct of health, the ADI, in the analyses.^47^ Social constructs are potentially important to consider in post-AFF fracture mortality analyses as social deprivation is a significant predictor of post-hip fracture mortality.^48^

We also acknowledge several limitations to this report. There were 38 men who had an ICD-9 or 10 code for a ST/FS fracture whose radiographs were not available for review. Veterans may receive care outside the VHA system, and these radiographs were not available.^49^ However, this is also a potential strength; by limiting the cohort to only those Veterans who received care at the VHA hospital system for their incident AFF, hip, or ST/FS fractures, differences in mortality following fractures that might be influenced by different payor systems^50^ would not have been a confounder. We used the 2014 ASBMR criteria^10^ for determining if a fracture was an AFF and required focal cortical hypertrophy at fracture origin and a primarily transverse fracture pattern, 2 key features recognized by a current task force in progress at the ASBMR, which may lead to future clarifications in the definition for AFF.^51^ In the statistical models, the proportional hazards assumption was violated for age at fracture, BMI, smoking status, VA-FI, prevalent fractures, and cumulative BP exposure. Therefore, we used time-dependent coefficients to account for non-proportional hazards. Information on ADI was only available starting at fiscal year 2007 and was missing for a number of men. Our data is confined to male Veterans who had received at least one filled prescription for a BP and may not be generalizable to non-Veterans or persons who had an AFF without any prior BP exposure. We could not determine if the prescribed BP was used for osteoporosis, as DXA data were not uniformly available and ICD codes for osteoporosis are underutilized.^52^ However, we did exclude men with ICD-coded metastatic cancer, metabolic bone diseases other than osteoporosis for which BPs might be prescribed, and those with more frequent dosing intervals for zoledronate, which may be used for cancer-related complications.^53^ Due to the small number of AFFs and a small number of mortality events among those with AFF, all comparative analyses involving this group were characterized by high variance. Consequently, the results did not provide sufficient statistical evidence to reliably differentiate the mortality risk of the AFF cohort from that of men with a hip fracture or an ST/FS fracture and note that these models are exploratory in nature. Our analyses on length of hospital stay, timing of hospital discharges, and post-fracture rehabilitation were post hoc. Veterans may have received post-fracture rehabilitation outside of the VHA, and we would not have captured this. Finally, this is an observational study, not a randomized clinical trial, and as such, is subject to confounding.^54^

In conclusion, both overall short-term (up to 1 yr) and overall long-term (up to 5 yr) mortality following an AFF in men treated with BPs for fracture prevention is not significantly different than mortality following either a hip or ST/FS fracture. However, it is possible that mortality is lower following an AFF than a hip or ST/FS fracture, but small sample sizes of AFF may have precluded ascertainment of such a difference. Future studies with larger numbers of men with AFF are needed to understand the risk-benefit ratio of BPs for fracture prevention in this population.

## Data Availability

The deidentified data may be available to the public and other VA and non-VA researchers with a Data Use Agreement under certain conditions which prioritize protecting Veteran’s privacy and confidentiality to the fullest extent possible.
